# The Revaluation of Plant-Derived Terpenes to Fight Antibiotic-Resistant Infections

**DOI:** 10.3390/antibiotics9060325

**Published:** 2020-06-13

**Authors:** Floriana Cappiello, Maria Rosa Loffredo, Cristina Del Plato, Silvia Cammarone, Bruno Casciaro, Deborah Quaglio, Maria Luisa Mangoni, Bruno Botta, Francesca Ghirga

**Affiliations:** 1 Laboratory affiliated to Pasteur Italia-Fondazione Cenci Bolognetti, Department of Biochemical Sciences, Sapienza University of Rome, P.le Aldo Moro 5, 00185 Rome, Italy; floriana.cappiello@uniroma1.it (F.C.); mariarosa.loffredo@uniroma1.it (M.R.L.); marialuisa.mangoni@uniroma1.it (M.L.M.); 2 Department of Chemistry and Technology of Drugs, “Department of Excellence 2018−2022”, Sapienza University of Rome, P.le Aldo Moro 5, 00185 Rome, Italy; cristina.delplato@uniroma1.it (C.D.P.); silvia.cammarone@uniroma1.it (S.C.); bruno.botta@uniroma1.it (B.B.); 3 Center For Life Nano Science@Sapienza, Istituto Italiano di Tecnologia, Viale Regina Elena 291, 00161 Rome, Italy; francesca.ghirga@iit.it

**Keywords:** antibiotic resistance, plant secondary metabolites, plant-derived natural products, terpenes, diterpenes, triterpenes, multidrug-resistant strains.

## Abstract

The discovery of antibiotics has revolutionized the medicine and treatment of microbial infections. However, the current scenario has highlighted the difficulties in marketing new antibiotics and an exponential increase in the appearance of resistant strains. On the other hand, research in the field of drug-discovery has revaluated the potential of natural products as a unique source for new biologically active molecules and scaffolds for the medicinal chemistry. In this review, we first contextualized the worldwide problem of antibiotic resistance and the importance that natural products of plant origin acquire as a source of new lead compounds. We then focused on terpenes and their potential development as antimicrobials, highlighting those studies that showed an activity against conventional antibiotic-resistant strains.

## 1. Introduction

The discovery of penicillin by Alexander Fleming (1881–1955) drastically revolutionized modern medicine and represented the beginning of the antibiotic era that saved millions of lives from bacterial infections [1]. Since then, according to the definition expressed by Selman Waksman, any small molecule, produced by a microbe, with antagonistic properties on the growth of other microbes was defined as “antibiotic” [2]. During the “Antibiotic Golden Age” (1940–1970), hundreds of molecules were isolated, characterized, classified and marketed [3,4,5]. For decades, antibiotics were used as “wonder drugs” to combat microbes but also as prophylactic agents in the field of agriculture, fish industry and breeding. The massive use, in addition to a high rate of prescriptions, mismanagement in the form of self-medication or interruption of therapy, led to an exponential appearance of resistant bacterial infections and consequent failure of antibiotic therapies [6,7]. This was followed by a post-antibiotic era where the discovery of new compounds is still in sharp decline, together with a proportional rise in the appearance of resistant strains [8,9,10,11,12]. The problem of antibiotic resistance is crucial and scientific research should not lag behind. Only in Europe, in fact, about 700 thousand cases of antibiotic-resistant infections provoke 33,000 deaths every year, with an estimated cost above €1.5 billion [13]. However, it should be considered that an estimated $200 million is required for a molecule to reach commercialization, with the possible risk of a rapid development of resistance and consequent interruption of clinical development [14]. Despite this, it is evident that new molecules with antimicrobial properties are urgently needed and nature represents the primary source of biologically active compounds, in agreement with Fleming’s statement: “I did not invent penicillin. Nature did that. I only discovered it by accident” [15]. In this review, we focus on the relevance of natural compounds from plants (plant-derived natural products, PDNPs) in the 21st century drug discovery, with emphasis to terpenes showing antimicrobial activities and that can represent promising candidates to fight multidrug-resistant (MDR) microbial infections.

## 2. Antimicrobial Resistance

Biologically active molecules are defined as antimicrobials if they are able to inhibit growth or to kill certain or various classes of microorganisms. However, microorganisms have developed several mechanisms to circumvent the action of these antimicrobial compounds. In this context, it is worthwhile to specify the difference between antimicrobial resistance and persistence. Resistance to a given molecule is maintained from the mother cell to the daughter cells, unless mutations make them susceptible again [16]. In comparison, persistence is defined as the ability of microbial cells to be recalcitrant to the antibiotic action, as they enter into a stationary phase of their growth (dormant cells). This leads to the inefficacy of the antibiotic agents since most of them act by inhibiting or interacting with specific metabolic processes that are not active in dormant cells [17,18]. It is also important to define the two major types of antimicrobial resistance: natural and acquired [19,20]. Natural resistance can be constantly expressed in the bacterial species (intrinsic resistance), whereas acquired resistance is expressed only upon exposure to an antimicrobial agent (induced resistance) [21]. The reduced permeability of the outer membrane and the activity of efflux pumps are classic examples of intrinsic resistance [22]. Acquired resistance occurs through acquisition of genetic material by means of transformation, conjugation, transposition (horizontal gene transfer) or by mutation in the chromosomal DNA [23,24]. Mutation of the drug target or in those genes involved in the regulation of drug transporters are examples of acquired resistance [19].

### Mechanisms of Antimicrobial Resistance

**Figure 1:**
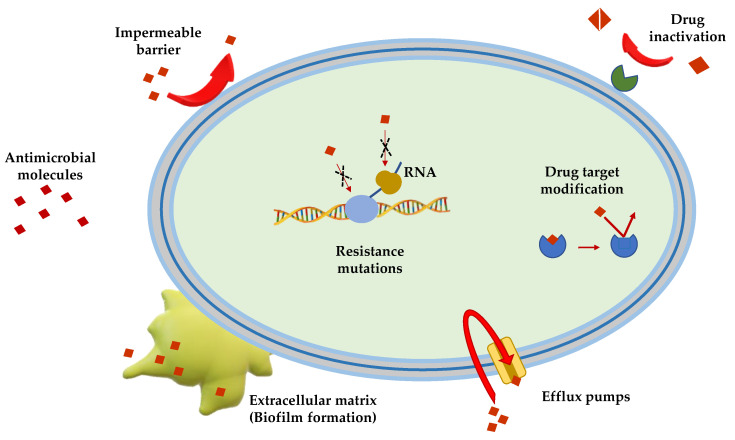
Schematic representation of the principal mechanisms of antibiotic resistance.

Gram(+) and Gram(−) bacteria possess and/or have developed several mechanisms of antimicrobial resistance that fall into five major categories (Figure 1):

*Limitation of drug uptake*. Bacteria can be intrinsically resistant to a certain antimicrobial due to their structure and morphology. Lipopolysaccharide (LPS) in Gram(−) bacteria, for example, provides a physical barrier that protects the cell from several groups of large molecules [25]. In these bacteria, drugs are internalized through porin channels that generally allow the uptake of hydrophilic molecules. Mutations that change their selectivity or that reduce the number of expressed porins are the two major mechanisms of antimicrobial resistance [26,27]. Gram(+) bacteria, lacking outer membrane, possess a peptidoglycan cell wall and the restricting drug uptake is not as prevalent. However, pathogenic bacterial species, i.e., *Staphylococcus aureus*, have developed a mechanism which consists in thickening the cell wall to limit the amount of drug that enters the cell [28,29]. *Mycoplasma* spp., devoid of cell wall, are intrinsically resistant to antimicrobials (e.g., β-lactams and glycopeptides) that interfere with cell wall synthesis and regulation [30].

*Drug inactivation.* Bacteria can produce several enzymes or molecules that inactivate drugs by covalent binding or enzymatic processes. Firstly, common antibiotics (e.g., aminoglycosides, streptogramins, fluoroquinolones, chloramphenicol) could be inactivated by acetylation, phosphorylation or adenylation; secondly, hydrolyzation is the primary mechanism by which bacteria can inactivate β-lactam antibiotics (e.g., cephalosporins, penicillins and cephamycins). β-lactamases are the most common example: these enzymes provide resistance to β-lactam antibiotics by hydrolyzing a specific site in the β-lactam ring structure [31]. Recently, β-lactamases were found to be active against carbapenems in Enterobacteriaceae (carbapenemases, i.e., *Klebsiella pneumoniae* carbapenemases and carbapenem-resistant enterobacteriaceae enzymes) [31].

*Mutation/alteration of the drug target.* The majority of antimicrobials have a specific mechanism of action against a specific cellular target and this is one of the reasons why bacteria are not susceptible to a certain class of molecules [32]. Gram(+) strains, for instance, become resistant to β-lactam drugs via alteration of the penicillin-binding proteins, that are transpeptidases involved in the cell wall construction [33]. *S. aureus* acquires resistance to the glycopeptide vancomycin by decreasing the binding ability of this molecule to the cell wall, as a consequence of a modification of the terminal d-Ala–d-Ala moiety of the peptidoglycan precursor lipid II [34].

*Drug efflux.* Bacteria can eliminate internalized toxic substances through a mechanism involving efflux pumps, which can be constitutively expressed or overexpressed under certain conditions. Many of these pumps have the capability to transport different types of substances. They are properly named multi-drug efflux pumps [35] and their increased number is generally associated to high-level of resistance to clinically significant bacterial infections [36,37].

*Biofilm formation.* In conditions of environmental stress, scarcity of nutrients, presence of antimicrobial molecules, some bacterial species can switch from a motile to a sessile lifestyle, named biofilm. This is a bacterial community able to colonize abiotic (e.g., medical devices and implants [38,39,40]) and biotic surfaces (e.g., human tissues [41,42,43]). Biofilm formation is a strategy used by pathogenic bacteria to protect themselves from the external stressful conditions by producing a thick and sticky extracellular matrix which contains DNA, proteins and polysaccharides. In addition, biofilm cells enter into a slow division rate, which weakens the effect of antibiotic molecules targeting specific cellular processes. Thus, biofilms are often associated to chronic infections and molecules capable to disrupt these communities and/or to inhibit their formation are highly demanded [44,45].

## 3. Plant Derived Natural Products

The practice of using plants for medicinal purposes is thousands of years old. Since the earliest civilizations, plants have played a major role in medicine due to their variability and abundance of therapeutic agents [46,47]. Notably, the first manuscript that reports the use of medicinal plants dates back to 2600 BC; it describes a complex medical system, with the use of over 1000 medicinal plants in Mesopotamia [48]. The “Ebers Papyrus” from about 1500 BC describes the usage of about 700 drugs (mainly plant-derived) in the Egyptian medicine, while traditional Chinese medicine has been extensively documented and nowadays it is still of reference [48,49,50]. In the West, the tradition of medicinal plants has foundations in the culture of Greek and Roman civilizations, as proved by the compendiums written by Dioscorides and Pliny the Elder, respectively [51]. However, until the 18th century AD, the use of plants in medicine has been based on empirical evidence. It was only with the first studies conducted by Anton von Störck on poisonous herbs (i.e., colchicum and aconite) and by William Withering on foxglove, that the foundations of a scientific research on medicinal plants were laid [52]. Rational drug discovery began in 1806 when Friedrich Sertürner isolated a bioactive compound from the poppy plant, i.e., the alkaloid with analgesic and sleep-inducing effects and that he called “morphine” in honor of the Greek God of dreams, Morpheus. This induced a significant boost in the research and isolation of bioactive compounds from plants, as evidenced by the numerous discoveries of the following decades. In 1820, Runge isolated caffeine from *Coffea arabica*; in 1824 the anti-tussive agent codeine was isolated from poppy by Robiquet, while in 1848 the anti-spasmodic alkaloid papaverine was identified by Merck Fraz; in 1869, digoxin (digitalis) was isolated from *Digitalis lanata* by Nativelle [53]. These discoveries led to an exponential growth of scientific and economic interest in the PDNPs which reaches up to the present day: ∼25% of the estimated 1.1 trillion US dollars invested in the pharmaceutical market annually, come from PDNPs [53]. Thanks to computational and biological approaches it has been easier to proceed with the identification, selection and production of PDNPs which nowadays possess a renewed scientific and economic potential [54,55].

### Plant-Derived Drug Discovery: The Two Sides of the Coin

Plant-derived drug discovery is currently hindered by a series of scientific, social and economic factors that deserve to be analyzed in order to contextualize the actual scenario of the pharmaceutical market.

*Socio-economic factors*. A main issue here is the access to starting materials. Initially, there is a process of correct identification and nomenclature that cannot be automated and requires experts in the field. These experts are also necessary for the correct collection, documentation and preparation of the investigated herbarium, but they are rather rare [56,57]. The isolation processes, albeit with high yield, lead to very low quantities of active compounds, thus requiring a very high amount of starting material. This is a crucial factor; many plants need precise habitats for their growth and optimal production of metabolites and have seasonal life cycles, making their harvest difficult to scale-up. Furthermore, the ecological balance of the belonging niche must be taken into consideration: in fact, it is important to evaluate the impact that a reduction in the number of plants in the niche can have on food chains and other living species. Finally, many plants grow only in certain regions and political factors must be taken into account for their harvest, such as wars, international relations between states, import/export laws [56].

*Scientific factors*. One of the most relevant concerns is undoubtedly the quality of the raw material. This is influenced by various conditions, including the time of harvest, the quality of the soil, possible environmental contamination as well as the type of processing and storage [58]. Biologically active compounds often have a complex chemical structure, with numerous chiral centers and substituents containing oxygen, which make their synthesis difficult in the laboratory. The extracts obtained from natural samples have a high viscosity, can precipitate or aggregate easily, but, above all, they often consist of a mixture of bioactive compounds that makes biological activity tests of dubious interpretation [59,60,61,62,63]. Another important issue is the mechanism of action that is not well defined yet, for some common compounds [64].

Besides the challenges mentioned above, PDNPs are considered promising candidates for the development of new drugs, thanks to their intrinsic characteristics.

First, they represent a direct source of therapeutic agents or phytomedicines. Between 1981 and 2014, the Food and Drug Administration (FDA) approved 1562 new drugs from natural sources, of which 141 (9%) were botanical mixtures and 64 (4%) were unaltered natural products [53]. According to recent analysis [49,53], the percentage of pharmacologically and phytochemically investigated plants is around 6% and 15% of the existing plant species, respectively. The plant kingdom can therefore be considered to be not fully explored and holds enormous potential. Furthermore, for some plants there is a well-documented millennial tradition of ethnopharmacology which represents an excellent starting point for more accurate scientific studies [65,66]. In addition, the advent of new techniques in separation, purification and characterization of novel compounds significantly improved the efficiency of these processes and, today, an important challenge is the generation of high-quality libraries of natural products that might allow the fast identification of lead compounds for drug discovery progression [67,68,69,70,71,72,73,74,75]. Importantly, PDNPs serve as scaffold for the synthesis of libraries with several chemical structures and for the design of lead compounds with a desired biological activity or markers for a specific detection [76,77,78]. These objectives can be achieved thanks to the contingent biological and computational approaches that can be used following one of these proposed models: the forward pharmacology or reverse pharmacology model. In the first case, the evidence of biological activity *in vivo* (i.e., antimicrobial assays, organ or tissue models, animal tests) are followed by in silico/in vitro analysis for target and mechanism identification. On the contrary, the more recent reverse pharmacology approach consists of an *in silico/in vitro* screening of a large libraries of compounds and the “hits” are selected for further *in vivo* characterization [53].

In this review we will focus on an important class of secondary metabolites derived from plants, i.e., terpenes, that are under investigation for their antimicrobial activity, especially against antibiotic-resistant strains. The data we report in the next paragraphs were obtained from research groups all over the world and summarize the in vitro antimicrobial activity of these compounds, highlighting their high potentiality as new antimicrobials. This class of PDNPs has already been studied and commercialized for other important human diseases as proved by the high number of clinical trials (Table 1) and marketed drugs (Table 2).

## 4. Terpenes

Terpenes are the most numerous and structurally diverse class of natural products. These compounds are characterized by different carbon skeletons, but, despite structural differences, all terpenes are unified by a common biosynthetic pathway: the fusion of five-carbon isoprene units, the basic structural unit of terpenes. Since the last century, the biosynthetic process of terpenes was explained by the isoprene rule, which states that all terpenes derive from the ordered head-to-tail joining of isoprene units. A head-to-tail fusion is the most common; however, non-head-to-tail condensation of isoprene units also occurs [90,91]. It is useful to divide terpenes into classes according to the number of isoprene units they are biogenetically made of. They are classified as hemiterpenes (one isoprenoid unit); monoterpenes (two isoprenoid unites); sesquiterpenes (three isoprenoid unites); diterpenes (four isoprenoid unites); sesteterpenes (five isoprenoid unites); triterpenes (six isoprenoid unites) and tetraterpenes (eight isoprenoid unites) [92]. As reported in the literature, several terpenes have been employed as important pharmaceutical agents with anti-inflammatory [93], anti-viral [94], anti-diabetic [95], anti-tumor [96] and antibacterial activities [97]. Notably, terpenes have played a pivotal role in the fight against antibiotic resistance showing promising antibacterial potential against multi-resistant strains or acting as potentiators for antimicrobials by exhibiting synergistic effects [98,99,100]. Due to their low yield from natural source, semi-synthetic and synthetic derivatives with improved biological properties have been developed [101,102]. The first step in studying medicinal plants is the preparation of plant samples to preserve the secondary metabolites prior to extraction. Compounds from different parts of a plant such as leaves, barks, roots, fruits and flowers can be extracted from fresh or dried plants material. Traditional methods such as maceration and Soxhlet extraction are widely employed in the studies reported in this review. First, the leaves are properly treated, dried and separated from foreign materials including soil, pebbles and other matters unsuitable for the solid-liquid extraction process. The fresh material is then air-dried, macerated usually by using a suitable solvent that, due to its polarity, is able to extract polar as well as less polar organic compounds. The obtained solution is the desired extract. In each case, extensive purifications of the obtained extract are performed by preparative Thin Layer Chromatography (TLC) or classic column chromatography or by advanced technology such as flash chromatography and High Performance Liquid Chromatography (HPLC) [103,104,105,106,107,108,109,110,111,112,113,114,115,116,117,118,119,120,121,122,123,124,125,126].

The structures and the purity of all terpenes reported in this review were unambiguously confirmed through nuclear magnetic resonance (NMR) spectroscopy, and by electrospray ionization mass spectrometry (MS) [103,104,105,106,107,108,109,110,111,112,113,114,115,116,117,118,119,120,121,122,123,124,125,126,127,128]. Most of the terpenes reported are known and their multiple chiral centers were assigned according to the literature. When chirality is not reported, terpenes were tested as racemic form (see Table 3) [103,107,126,129]. In some studies, the authors investigated the extract chemical composition by Gas Chromatography-mass spectrometry (GC/MS) [129]. The chemical structure of the most representative terpenes examined in this review is shown in Table 3.

### 4.1. Monoterpenes

Monoterpenes are the main constituents of essential oils and they are responsible of the flavor and aroma of plant from which they are extracted. They are formed by the dimerization of isoprene units and, based on the arrangement of their carbon skeleton, they are grouped into acyclic and cyclic structures [132]. It is also useful to classify monoterpenes in line with their different chemical functionalities, including alcohol (such as linalool and geraniol), aldehyde (such as citral and citronellal), phenol (such as thymol and carvacrol), ketone (such as carvone and camphor), ether (such as eucalyptol) and hydrocarbon (such as cymene, pinene, limonene, and phellandrene) groups [92]. It has been long recognized that monoterpenes possess antimicrobial activity. Griffin et al. investigated the relationships between the structure/molecular properties and the antimicrobial activity of terpenes. They found that hydrogen bonding parameters are associated with their biological activity, in all cases [133]. Several oxygenate monoterpenes such as carvacrol, thymol, menthol, and geraniol exerted antimicrobial activity against several Gram(+) and Gram(−) bacteria [134]. In particular, geraniol (Table 3, compound **1**), an acyclic monoterpene featuring alcoholic functionalization, has been extensively studied for its promising antimicrobial activities against MDR strains. Lorenzi et al. reported the ability of the essential oil, obtained from *Helichrysum italicum*, to significantly reduce the resistance of three pathogenic MDR Gram(−) bacteria to chloramphenicol [130]. The authors demonstrated the ability of the *H. italicum* essential oil to reduce chloramphenicol resistance of an *Enterobacter aerogenes* strain EAEP289 that over-expresses its AcrAB efflux pump, supporting their hypothesis regarding a potential efflux pump inhibitor (EPI) as a mechanism of antimicrobial activity. Further investigations confirmed that the essential oil derived from *H. italicum* contains one or more chemical constituents with EPI activity. Accordingly, chloramphenicol susceptibility testing was performed in the presence of several components of the most active fraction from the essential oil and, among them, geraniol resulted to be the most potent compound in reducing chloramphenicol minimum growth inhibitory concentration (MIC) up to 16-fold for the EAEP289 strain [130]. In addition, geraniol completely reversed the chloramphenicol resistance in combination with the well-studied EPI, phenylalanine arginine β-naphthylamide (PAβN) control. Interestingly, the efficiency of geraniol in reducing drug resistance was also observed towards β-lactams and the fluoroquinolone norfloxacin highlighting that geraniol modulates antibiotic resistance in Gram(−) bacteria by targeting efflux pumps mechanisms [130]. Recently, Sayout et al. evaluated the antibacterial activity and the chemical composition of the essential oils from two species of the genus Lavandula: *L. tenuisecta* Coss.exBall and *Lavandula pedunculata* subsp.atlantica (BRAUN-BLANQ) [129,135]. Essential oils from both species showed antibacterial activity against several microorganisms leading to an increased interest for Lavandula genus in the treatment of bacterial infections caused by MDR strains. The two species are rich of oxygenated monoterpenes and the major constituents are camphor, fenchone and 1,8-cineole. The authors investigated the relationship between terpenoid constituents and antibacterial activity in order to identify the compound(s) that are responsible for the antibacterial activity of *Lavandula atlantica* essential oil. Among them, one of the major constituents, camphor (Table 3, compound **2**), a bicyclic monoterpene ketone, exhibited antimicrobial activity against all the tested strains. Several other oxygenated monoterpenes isolated from *Lavandula atlantica* essential oil showed a strong antimicrobial activity against most of the studied strains. Among them, linalool (Table 3, compound **3**), an acyclic monoterpene alcohol, with MIC values ranging from 1.44 μg/mL to 3.83 μg/mL; terpinen-4-ol (Table 3, compound **4**), a cyclic monoterpene alcohol, with MIC values ranging from 0.78 μg/mL to 3.13-6.25 μg/mL; borneol (Table 3, compound **5**), a bicyclic monoterpene alcohol, with MIC values ranging from 0.47 μg/mL to 3.75 μg/mL; and fenchone (Table 3, compound **6**), a bicyclic monoterpene ketone, with MIC values ranging from 1.06 μg/mL to 4.25 μg/mL [129,135].

### 4.2. Sesquiterpenes

Sesquiterpenes are composed of three isoprene units and they are widely studied for their biological activities. Farnesol is a compound of 15 carbon atoms and can be considered the precursor of acyclic sesquiterpenes. This sesquiterpene, has shown a significant antimicrobial activity against several bacteria, including *Staphylococcus aureus* and *S. epidermidis*, with the ability to inhibit biofilm formation of *Streptococcus* spp. [134]. Furthermore, it was found to potentiate the activity of β-lactam antibiotics against antibiotic-resistant bacteria [136]. Recently, Kim et al. reported that two synthetic derivatives of farnesol (Table 3, compounds **7** and **8**) had an antimicrobial activity against three different methicillin-resistant *S. aureus* (MRSA) with MICs of 512 μg/mL and 256–512 μg/mL for compounds **8** and **7**, respectively). In addition, they displayed an enhancer effect of β-lactam antibiotics. For instance, when used in combination with oxacillin against resistant strains, farnesol and their derivatives were able to reduce the oxacillin MIC of up to 128-fold. A lower, but still significant potentiating activity was also observed in combination with ampicillin [127]. Moreover, by cyclization reactions a wide variety of monocyclic, bicyclic and tricyclic compounds can be formed [132]. Lee et al. investigated the relationship between the sesquiterpene lactone structure and the antimicrobial activity. The authors demonstrated that the activity depends on the presence of a beta unsubstituted cyclopentenone ring moiety and how its saturation dramatically reduced activity [137]. Several compounds belonging to the largest class of sesquiterpene lactones, the pseudo-guaianolides were also identified [138]. Among them, Arnicolide D and Arnicolide C (Table 3, compounds **9** and **10**), isolated from *Centipeda minima*, a medicinal plants of Nepal, showed activity against both MRSA and methicillin sensitive *S. aureus* (MSSA) with MIC values of 300 μg/mL *versus* MRSA, and 75 μg/mL and 38 μg/mL *versus* MSSA, respectively [103]. Furthermore, a related guaianolide 5 (Table 3, compound **11**), isolated from *Artemisia gilvescens* manifested an excellent potential against a clinical strain of MRSA with a MIC value of 1.95 μg/mL [104]. Ordónez et al. studied the antimicrobial activity of two sesquiterpene lactones, belonging to the guainolide group, isolated from *Gynoxys verrucosa*, against six clinical isolates of *S. aureus* and *S. epidermidis* with different drug-resistance profiles. The sesquiterpene dehydroleucodine (Table 3, compound **12**) exhibited antimicrobial activity against all staphylococcal isolates, including four methicillin-resistant strains with a minimum concentration inhibiting 50% bacterial growth (IC_50_) between 49 and 195 μg/mL. Interestingly, the antimicrobial activity of dehydroleucodine and other structurally related sesquiterpene lactones, such as Arnicolide C, against MRSA and MSSA strains, suggested that the presence of a carbonyl moiety in the opposite side of the cycloheptane ring acts as a secondary hydrogen binding point and that the methylene group in the lactone ring significantly affects antimicrobial activity [105]. Among the phenolic sesquiterpenes, Xanthorrizol (Table 3, compound **13**) from *Cinnamomum iners*, displayed significant antibacterial activity against MRSA with a MIC of 25 μg/mL [106]. Goncalves et al. inquired into the antibacterial effect of a wide range of antibiotics, such as tetracycline, erythromycin, penicillin, and vancomycin, when combined with nine sesquiterpenic compounds, e.g., hydrocarbons and alcohols, on two clinically relevant *S. aureus* and *Escherichia coli* strains with well-defined resistance-sensitive profiles. Several combinations of antibiotic-sesquiterpenic compounds increased the antibacterial activity of the antibiotics against *S. aureus*. For *E. coli*, an antagonistic effect was observed. Moreover, this study paved the way for the evaluation of sesquiterpenes as possible antibiotic enhancer against MRSA [139].

### 4.3. Diterpenes

Diterpenes consist of a chemically diverse group of compounds, all with a C20 carbon skeleton based on four isoprene units. They can be classified as linear, bicyclic, tricyclic, tetracyclic, pentacyclic or macrocyclic diterpenes depending on their skeletal core. A great number of diterpenes showed significant antimicrobial activity against MDR bacteria, and/or the ability to enhance the effectiveness of antibiotics when evaluated in combination with them against resistant strains [90,134,140]. Labdane-type bicyclic diterpenes, featuring a decalin system and a six-member ring which may be open or closed with an oxygen atom, proved an interesting antimicrobial activity against MDR strains [102]. Six new labdane-type diterpenes, isolated from the Malaysian species of *Vitex vestita* were tested for the first time against a panel of 46 Gram(+) bacterial strains, both sensitive or resistant to conventional antibiotics. Interestingly, vitexolide A (Table 3, compound **14**) resulted the most active compound with MICs ranging from 2 to 32 μg/mL against all tested microorganisms and a MIC of 4 μg/mL against the human MDR *S. aureus* strain CRBIP 21.21. Acumenolide (Table 3, compound **15**) indicated an anti-*Bacillus* activity (MIC of 16 μg/mL) while 12-epivitexolide A (Table 3, compound **16**), the C12 epimer of vitexolide A, and acuminolide, showed moderate antibacterial activity against a panel of *S. aureus* strains with MIC values of 16 μg/mL. The structure-activity relationship analysis underlined that the presence of the γ- hydroxybutenolide moiety along with a C12 hydroxylation are essential for the antimicrobial activity [107]. Interestingly, another bicyclic diterpene with labdane-type scaffold, the 8(17),12E,14-labdatrien-6,19-olide (Table 3, compound **17**), featuring a five-member lactone ring and three unsaturations, was isolated from *Salvia leriifolia* and showed a significant antimicrobial activity against a clinical MRSA strain with a MIC of 213 µM [108]. A structurally related diterpene, 8(17),11(Z),13(E)-trien-15,19-dioic acid (Table 3, compound **18**), isolated from *Caesalpinia decapetala* displayed a moderate antibacterial activity against a MRSA strain with a IC_50_ of 5.99 µg/mL [109]. Recently, Siddique et al. examined the antibacterial activity of a labdane diterpene, the (E)-8(17),12-labdadiene-15,16-dial (Table 3, compound **19**) from *Zingiber montanum*, against a panel of clinical isolates of MDR *S. aureus* and MRSA and found out a potent activity with MICs ranging from 46 to 93 µg/mL. In addition, the authors pointed out that the unsaturations (exomethylene C-8 and olefine C-12) and the two aldehyde groups at C-16 and C-17 on the diterpenoid’s carbon skeleton could account for the significant activity against MRSA strains [110]. Gupta et al. reported the antimicrobial activity of a clerodane diterpene, 16α-hydroxycleroda-3, 13 (14)-*Z*-dien-15, 16-olide (CD) (Table 3, compound **20**), isolated from leaves of *Polyathia longifolia*, against seven clinical MRSA isolates with MICs in the range of 15.625–31.25 μg/mL (15.625 μg/mL against reference strain SA-96) [128]. The authors also investigated the synergistic interactions and the resistance-modifying potential of CD in combination with fluoroquinolones against these strains and its efflux pump inhibitory potential [141]. This diterpenoid enhanced the efficacy of norfloxacin, ciprofloxacin and ofloxacin against MRSA clinical isolates reducing up to 16-fold the MIC of norfloxacin against the MRSA clinical isolate ST2071. Furthermore, the results of time-kill kinetics revealed that CD in combination with norfloxacin at their ½MIC significantly reduced the viability of bacterial cells while flow cytometric analysis and spectrofluorometric assay clearly indicated that CD diterpene inhibited an MDR efflux pump [141]. The abietane-type is a class of tricyclic diterpenoid widespread in several botanical families. The aromatic abietanes are the largest group of naturally occurring abietanes. They are characterized by an aromatic C ring and a different degree of oxygenation at several positions. Generally, aromatic abietanes are not functionalized on the A-ring carbons. Most of them presents a different degree of oxidation in their B- and C-ring carbons [142]. Gaspar-Marques C. et al., investigated the antibacterial effect of several natural abietanes isolated from *Plectranthus grandidentatus* and *P. hereroensis* against MRSA and vancomycin-resistant *Enterococcus faecalis* (VRE). Diterpenes coleon U, 7α-acetoxy-6β-hydroxyroyleanone and horminone (Table 3, compound **21**, **22** and **23**) were the most active, with a remarkable activity against MRSA strains (MICs ranging from 0.98 µg/mL to 15.63 µg/mL) and a more moderate activity against VRE strains (MIC values from 15.63 and 31.25 µg/mL) [111]. Among the other abietanes tested, diterpenes 7α,12-dihydroxy-17(15-16)abeo-abieta-8,12,16-triene-11,14-dione (Table 3, compound **24**) and 16-acetoxy-7α,12-dihydroxy-8,12-abietadiene-11,14-dione (Table 3, compound **25**) showed a weaker activity, with MICs comprised between 15.63 µg/mL and 62.50 µg/mL. The authors suggested that 12-hydroxy-p-benzoquinone moiety in ring C together with an oxidized B ring at the C-6 and C-7 positions are essential for antimicrobial activity against MRSA and VRE. Furthermore, the most promising compound, coleon U, consists of the more oxygenated and dehydrogenated chromophoric system through rings B and C. Moreover, the presence of the 6β-hydroxyl and 7α-acetoxy groups, as in 7α-acetoxy-6β-hydroxyroyleanone, or the oxidation at C7 position, as in horminone and abietanes 24 and 25, preserved a good antimicrobial activity [111]. Recently, Jurkaninovà S. and co-workers evaluated the anti-MRSA activity of abietane diterpenes from *Coleus blumei* Benth. Sincoetsin C (Table 3, compound **26**) was the most potent compound with a MIC of 128 µg/mL against the MRSA strain CCM 4750 whereas the abietane glucosides did not show a valuable growth inhibition of MRSA, suggesting that the glycosylation of the hydroxyl group on the abietane skeleton suppresses the anti-MRSA activity [112]. Notably, Porto et al. studied the structure-antimicrobial activity relationships of pimarane-type diterpenes against MDRs, increasing the diversity of chemical structures by microbial transformation. Five tricyclic diterpene derivatives of the ent-8(14), 15-pimaradiene (PD), isolated from *Viguiera arenaria*, were obtained by fungal transformation in *Aspergillus ochraceus*. This allowed to introduce a reactive center into the poorly functionalized PD skeleton which was then tested against eight clinically isolated MDR bacteria. Interestingly, the diterpene derivative ent-8 (14), 15-pimaradien-3β-ol (Table 3, compound **27**) was much more effective than its biosynthetic precursor, with promising MIC values (lower than 10.0 μg/mL) against a large variety of microbial pathogens. The time-kill curve experiments were carried out on MDR strain of *S. aureus* and showed that this compound was endowed with bactericidal action at all the evaluated concentrations (8.0, 16.0 and 24.0 µg/mL) within 24 h of incubation. Additionally, it was tested in combination with vancomycin at the minimal bactericidal concentration. Time-kill curve profile of resulting combination denoted that the number of viable microorganisms was drastically reduced within the first six hours. Furthermore, by comparing structural features of the tested compounds with biological data, the authors established that the antimicrobial activity of pimarane-type diterpenes was ruled by structural factors other than the hydrogen-bond-donor (HDB) Urzúa hypothesis suggesting that the structural requirements responsible for an efficient antibacterial activity include a lipophilic structure, capable of insertion into the cell membrane and one hydrophilic group (HBD) that interacts with the phosphorylated headgroups of membrane phospholipids [113,143]. In 2009, Stavri and co-workers analyzed the antimicrobial activity of three new diterpenes, isolated from *Plectranthus ernstii*, against a panel of MRSA. Among them, the pimarane diterpene rel-15(ζ),16-epoxy-7R- hydroxypimar-8,14-ene (Table 3, compound **28**), exhibited moderate antistaphylococcal activity against MDR strains with a MIC of 32 µg/mL, whereas rel-15(ζ),16-epoxy-7-oxopimar-8,14-ene and 1R,11S-dihydroxy-8R,13R-epoxylabd-14-ene (Table 3, compound **29** and **30**) showed a weaker activity. Surprisingly, a simple structural difference between the two pimarane diterpenes from 7-hydroxy to 7-oxo in the pimarane compound resulted in a loss of antistaphylococcal activity, presumably due to an increased lipophilicity and poorer uptake of the compound [114]. Among the pimarane-type diterpenes, Smith et al. reported the antimicrobial activity of isopimaric acid (Table 3, compound **31**), from *Pinus nigra*, against strains with MDR efflux pumps and epidemic MRSA with MIC from 32 μg/mL to 64 μg/mL [115]. The authors also evaluated the activity of isopimaric acid in combination with tetracycline, norfloxacin and erythromycin or ofloxacin against the corresponding strains. However, no reduction of MIC was obtained for any of the antibiotics. Interestingly, isopimaric acid tested in combination with the efflux pump inhibitor reserpine (20 μg/mL) against two resistant strains, significantly increased the MIC for this compound. The authors postulated that isopimaric acid is not a substrate for these efflux pumps, but it is also possible that an antagonistic interaction with reserpine may render the antibiotics inactive. A molecular modeling study, performed by Zloh et al., reported the possible formation of complexes between MDR inhibitors and the substrates of efflux pumps. This would facilitate the entry of MDR inhibitors into the cell, following by complex dissociation, to allow the drug displaying its effect [144]. Based on this finding, it is possible that the complex formation is due to strong interactions, with a very slow rate of dissociation. This would lower the concentration of the free drug thus decreasing its anti-bacterial activity. Further investigations by Smith et al. supported this hypothesis highlighting a possible complex formation between diterpenes and reserpine responsible for a reduction in activity [115]. Recently, Soares A.C. and co-workers analyzed the antibacterial activity of two natural diterpenes against a set of seven clinically isolated MRD bacteria, including three *S. aureus* strains [116]. The two diterpenes, the tetracyclic *ent*-kaurenoic acid and the tricyclic ent-pimaradienoic acid (Table 3, compound **32** and **33**), featuring a different basic skeleton, were isolated from *Mikania glomerata* and *Viguiera arenaria*, respectively, and selected for this study according to the Urzúa hypothesis. The biological assays gave MIC values lower than 13 µg/mL, supporting the HBD Urzúa hypothesis. In order to perform structure-activity relationships studies the authors developed several semi-synthetic derivatives from both natural diterpenes. The hydrogenated product derivative of 33 (Table 3, compound **34**), resulted to be even more efficient as an antimicrobial agent against MRSA and *S. capitis* with a MIC of 6.25 μg/mL [116]. Furthermore, Barbosa et al. reported the potent antibacterial activity of the *ent*-kaurenoic acid isolated from *C. reticulata* oleoresin against MRSA (IC_50_ 3.4 µg/mL) [117]. Interestingly, a docking-based virtual screening of an *in-house* library of natural products within the catalytic site of ArnT, the enzyme responsible for colistin resistance mediated by lipid A aminoarabinosylation in *Pseudomonas aeruginosa*, led to the identification of the ent-beyer-15-en-18-O-oxalate (BBN149, Table 3, compound **35**), as promising inhibitor of ArnT [118]. This natural tetracyclic diterpene, featuring the *ent*-beyerene scaffold, was isolated from *Fabiana densa* var. *ramulosa*. The compound demonstrated to act as potent colistin adjuvant, potentiating its antibacterial activity up to 16-fold against colistin-resistant *P. aeruginosa* and *K. pneumoniae* isolates. The compound showed no activity against colistin-susceptible strains and no relevant toxicity towards human cells. The binding mode of BBN149 within the catalytic site of ArnT was evaluated by molecular docking simulations and identified two main binding poses. In both poses, the oxalyl group nicely overlaps with the crystallographic phosphate moiety and establishes H-bond interaction with the Lys85 residue, which was shown to be important for ArnT activity. Notably, this new insight made this diterpene a promising candidate for lead optimization of colistin resistance inhibitors with improved activity and/or pharmacological properties. Furthermore, natural analogs of the initial hit compound were isolated, and several semisynthetic analogs were designed and synthesized to afford structure-activity relationships (SAR). Currently, these data are covered by a patent [145].

### 4.4. Triterpenes

Triterpenes are composed by a carbon skeleton of six isoprene units and the main groups are represented by tetracyclic and pentacyclic structures. Pentacyclic triterpenes are all based on a 30-carbons skeleton comprising five six-membered rings (such as ursanes and lanostanes) or four six-membered rings and one five-membered ring (such as lupanes and hopanes). Interestingly, several pentacyclic triterpenes have been described for their antimicrobial activity [146,147]. Chung et al. reported the antimicrobial activity of α-amyrin, betulinic acid and betulinaldehyde (Table 3, compound **36**, **37** and **38**) against clinical isolates of MRSA and MSSA with MICs ranging from 64 to 512 μg/mL [119]. These pentacyclic triterpenes featured five six-membered rings, in the case of α-amyrin, or four six-membered rings and one five-membered ring, for betulinic acid and betulinaldehyde. Noteworthy, although their MICs were higher than those of the commonly used antibiotics vancomycin and methicillin, the combinations of these drugs with such pentacyclic triterpenes reduced the MIC to a range of 0.05 to 50%. Interestingly, the authors also demonstrated the potential synergistic activity of the standard antibiotics with these diterpenes on MRSA and MSSA [119]. Horiuchi et al. identify oleanolic acid, a pentacyclic triterpenoid related to betulinic acid, as the compound responsible for the antimicrobial activity of the *Salvia officinalis* (Sage) leaves extract against VRE. This pentacyclic triterpene (Table 3, compound **39**), an olean-12-en-28-oic acid substituted by a beta-hydroxy group at position 3, displayed a good activity against VRE and MRSA strains with MIC values of 8 µg/mL and 16 µg/mL, respectively. To get insight into structure-antimicrobial activity relationships, the authors tested structural related triterpenes and, among them, ursolic acid (Table 3, compound **40**), the C19 epimer of oleanolic acid. It showed a higher antimicrobial activity against both VRE and MRSA strains with MIC values of 4 µg/mL and 8 µg/mL, respectively [120]. Furthermore, ursolic acid, isolated from *Eucalyptus tereticornis*, did not exhibit antibacterial activity against the nalidixic acid-sensitive (NASEC) and nalidixic acid-resistant (NAREC) strains of *E. coli*, but in combination with nalidixic acid, this pentacyclic triterpene reduced the MIC of drug by two-fold (MIC of nalidixic acid alone was 6.25 µg/mL on NASEC and 100 µg/mL against NAREC). Based on these results, several semisynthetic derivatives of ursolic acid were developed and tested to evaluate their drug resistance reversal potential. Among them, the butyl and isopropyl ester of ursolic acid (Table 3, compound **41** and **42**) reduced the MIC of nalidixic acid by 8-fold against NAREC and by 4- to 8-fold against NASEC [121]. Furthermore, in a study reported by Wang et al. on the antibacterial and synergistic activity of pentacyclic triterpenes isolated from *Alstonia scholaris*, ursolic acid showed a good antimicrobial activity against Gram(+) pathogens including a MRSA strain, with a MIC value of 64 µg/mL and a synergistic effect with ampicillin and tetracycline against this strain [122]. A preliminary screening of a 350 compound proprietary in-house library containing marketed drugs and natural compounds to identify small molecules able to potentiate the antibacterial activity of some antibiotics against MRSA strains, led to the selection of 18β-glycyrrhetinic acid (18β-GA, Table 3, compound **43**), as the most active compound against *S. aureus* ATCC6538. It is a pentacyclic triterpene featuring five six-membered rings and found in nature together with its 18α-epimer (18α-GA) as aglycons of glycyrrhizin in *Glycyrrhiza glabra*. The authors discovered a synergistic effect of 18β-GA with several antibiotics against the MRSA strain LUH14616. Notably, this triterpene also potentiated the activity of tobramycin (16–32-fold), amikacin (8-fold) and polymyxin B (32–64-fold) against LUH21416, indicating 18β-GA as a good antibiotic enhancer against MRSA [131]. Recently, the lupane triterpene 3β,6β,16β-trihydroxylup-20(29)-ene (CLF1, Table 3, compound **44**), isolated from *Combretum leprosum* Mart. leaves, showed antibacterial action with clinical relevance against 10 MDR strains with MIC ≥ 4 µg/mL and, more interestingly, synergistic effects were observed in combination with the antibiotic amikacin, reducing the MIC value from 100 µg/mL to 80 µg/mL. In addition, this pentacyclic triterpene increased antimicrobial activity against MDR *E. coli* when associated with the antibiotics gentamicin and amikacin lowering MIC values from 20 µg/mL to 15 µg/mL and from 100 µg/mL to 60 µg/mL [123]. Noteworthy, the triterpenes with cycloartane skeleton were active against the antibiotic resistant clinical isolates [148]. These triterpenes have a unique pentacyclic structure characterized by cyclopropyl methylene group on the carbon skeleton. In a study reported by Gutierrez-Lugo et al., three cycloartane-type triterpenes i.e., 16R-hydroxymollic, 15R-hydroxymollic and 7β,16β-dihydroxy-1,23-dideoxyjessic acids (Table 3, compounds **45**, **46** and **47**), isolated from the aerial parts of *Acalypha communis*, exhibited moderate antimicrobial activity against VRE with MIC values of 8 μg/mL, 32 μg/mL and 8 μg/mL, respectively. In addition, 45 also displayed moderate activity against MRSA (MIC 64 µg/mL) [124]. Furthermore, Wang et al. evaluated the antimicrobial activity of new five cycloartane-type triterpenes, isolated from *Acacia grandifolia*, against two MRSA strains. These compounds consisted in three new 29-nor-cycloart triterpenes aphagrandinoids A–C, and a new natural product, aphagrandinoid D (Table 3, compound **48**), along with a structurally related known one (20R)-3β-hydroxy-24,25,26,27-tetranor-5α-cycloartan-23,21-olid. Among these compounds, the last triterpene, belonging to the 3β-cycloartanol series, showed the strongest antibacterial activity against MRSA strains (MIC values of 1.57 μg/mL *versus* 25 or 50 μg/mL), despite a slightly weaker antimicrobial potency compared to the commercial drug vancomycin (MIC of 0.78 μg/mL) [122]. Several triterpenes with tetracyclic structures were also found to be active against MDR strains. In line with this, Mosa et al. evaluated the antibacterial activity of two triterpenes i.e., 3β-hydroxylanosta-9, 24-dien-21-oic acid and methyl-3β-hydroxylanosta9,24-dienoate (Table 3, compound **49** and **50**), isolated from *Protorhus longifolia*, against a panel of antibiotic resistant Gram(+) and Gram(−) bacteria. The triterpenes had an antibacterial activity comparable to that of conventional antibiotics against resistant strains of *S. aureus* [126].

## 5. Conclusions

Despite the discovery of antibiotics that has saved millions of lives from microbial infections, the latter continue to be a serious threat to public health due to the appearance of resistant strains. New molecules with antimicrobial activities are therefore urgently needed. Plant-derived terpenes represent an interesting class of molecules with a multitude of activities that make them of reference for drug-discovery as proven by the numerous clinical trials and drugs on the market ( Table 1; Table 2). Terpenes also possess antimicrobial activity and, in recent years, the studies we have reported have shown their ability to act also against antibiotic-resistant strains. This class of compounds therefore has all the potential for the development of new antimicrobials, especially in this post-antibiotic era in which we are living.

## Figures and Tables

**Table 1:** Plant-derived terpenes in clinical trials (CT). Includes Not yet recruiting, Recruiting, Enrolling by invitation, Active not recruiting, Suspended and Completed trials of these compounds, parent extracts, or derivatives, applied alone or as a mixture with other constituents. Determined from www.clinicaltrials.gov on 14 May 2020.

Compound	Plant Source	Number of CT	Medicinal Purposes	Ref.
Andrographolide	*Andrographis paniculata*	7	Squamous Cell Carcinoma of Esophagus, Multiple Sclerosis, Cognitive Impairment, Migraine Disorders, Arthritis Rheumatoid, Acute Respiratory Infections.	[79]
Artemisinin	*Artemisia annua* L.	161	Schizophrenia, Malaria, Plasmodium Falciparum Malaria, Malaria in Pregnancy, Uncomplicated Falciparum Malaria, Vivax Malaria, Human Papilloma Virus Infection, Pre-Cancerous Dysplasia, Cervical Dysplasia, Metastatic Breast Cancer, Cytomegalovirus Infections, Increased Drug Resistance, Colorectal Cancer, Bowel Cancer, HIV, G6PD Deficiency, *Schistosoma Haematobium* and *Schistosoma Mansoni*.	[64,80,81]
Betulin	*Betula* spp.	4	Epidermolysis Bullosa, Wounds, Burns.	[82]
Betulinic acid	*Betula pubescens, Hypericum lanceolatum*	2	Dysplastic Nevus Syndrome, Anxiety, Psychological Stress.	[82,83]
Boswellic acid	*Boswellia serrata*	4	Relapsing Remitting Multiple Sclerosis, Renal Stones, Knee Osteoarthritis, Joint Pain, Stiffness.	[79]
Geraniol	*Cinnamomum* *tenuipilum, Valeriana officinalis*	2	Coronary Artery Disease, Uterine Cervical Dysplasia, Papillomavirus Infections.	[84]
Ginkgolides	*Ginkgo biloba*	7	Intravenous Alteplase Thrombolysis, Neurological Improving, Allergy, Ischemic Stroke.	[85]
Gossypol	*Gossypium hirsutum* L.	21	Recurrent Adrenocortical Carcinoma, Stage III and IV Adrenocortical Carcinoma, Extensive Stage Small Cell Lung Cancer, Unspecified Adult Solid Tumor, Adult Glioblastoma, Adult Gliosarcoma, Recurrent Adult Brain Tumor, Chronic Lymphocytic Leukemia, Recurrent Chronic Lymphocytic Leukemia, Follicular Lymphoma, Adenocarcinoma of the Prostate, Prostate Cancer, Diffuse Large Cell Lymphoma, Mantle Cell Lymphoma, Laryngeal Cancer, Brain and Central Nervous System Tumors.	[80]
Limonene	*Citrus* spp., *Apium graveolens*	10	Breast Cancer, Allergic Contact Dermatitis Due to Cosmetics, Obesity.	[86]
Lupeol	*Hymenocardia acida*	2	Acne.	[83]
Triptolide	*Tripterygium wilfordii*	4	HIV-infection/AIDS, Advanced Cancer, Gastric Cancer, Breast Cancer, Pancreatic Cancer, Prostate Cancer, Metastatic Colorectal Cancer, Solid Tumor, Solid Carcinoma, Solid Carcinoma of Stomach, Cancer of Stomach.	[64,85,87]
Ursolic acid	*Rosmarinus officinalis, Malus domestica, Salvia officinalis, Thymus vulgaris*	2	Metabolic Syndrome X, Sarcopenia.	[88]

**Table 2:** Plant-derived terpenes approved for therapeutic use. Additional resources from https://www.drugs.com/ and https://www.drugbank.ca/

Compound	Trade Name	Plant Source	Medicinal Purposes	Ref.
Arglabin	Arglabin	*Artemisia glabella*	Cancer chemotherapy.	[80,81]
Artemisinin	Artemisinin	*Artemisia annua* L.	Antiprotozoal agent: Antimalarial.	[80,81]
Docetaxel	Taxotere	*Taxus baccata*	Treatment of head, neck, stomach, lung, prostate, breast and ovarian cancers.	[79,81]
Ingenol mebutate	Picato	*Euphorbia peplus* L.	Actinic keratosis.	[80,87]
Paclitaxel	Taxol^®^, Paxene^®^, Abraxane, Nanoxel	*Taxus brevifolia* Nutt.	Chemotherapeutic agent for many types of cancer.	[80,89]

**Table 3:** Summary of triterpenes with antimicrobial activity against resistant strains.

N.	Common Name	Chemical Structure	Tested Microorganism	Antimicrobial Effect	Source	Ref.
**Monoterpenes**						
1	Geraniol	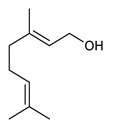	*E. aerogenes*	Efflux Pump Inhibitor	Species: *Helichrysum italicum*	[130]
2	Camphor	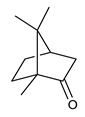	*S. aureus* *E. coli* *E. aerogenes* *P. aeruginosa* *K. pneumoniae* *K. oxytoca* *Salmonella* spp. *A. baumannii* *E. cloacae*	Growth inhibition	Species: *Lavandula pedunculata subsp.atlantica* (BRAUN-BLANQ)	[129]
3	Linalool	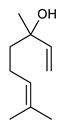	*S. aureus* *E. coli* *P. aeruginosa* *K. pneumoniae* *K. oxytoca* *Salmonella* spp. *A. baumannii* *E. cloacae*	Growth inhibition	Species: *Lavandula pedunculata subsp.atlantica* (BRAUN-BLANQ)	[129]
4	Terpinen-4-ol	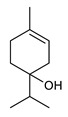	*S. aureus* *E. coli* *P. aeruginosa* *K. pneumoniae* *K. oxytoca* *Salmonella* spp. *A. baumannii* *E. cloacae*	Growth inhibition	Species: *Lavandula pedunculata subsp.atlantica* (BRAUN-BLANQ)	[129]
5	Borneol	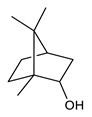	*S. aureus* *E. coli* *E. aerogenes* *P. aeruginosa* *K. pneumoniae* *K. oxytoca* *A. baumannii* *E. cloacae*	Growth inhibition	Species: *Lavandula pedunculata subsp.atlantica* (BRAUN-BLANQ)	[129]
6	Fenchone	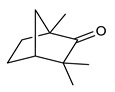	*S. aureus* *E. coli* *E. aerogenes* *P. aeruginosa* *K. pneumoniae* *K. oxytoca* *Salmonella* spp. *A. baumannii* *E. cloacae*	Growth inhibition	Species: *Lavandula pedunculata subsp.atlantica* (BRAUN-BLANQ)	[129]
**Sesquiterpenes**						
7	Farnesyl amine 2	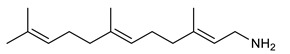	*S.aureus*	Growth inhibition		[127]
8	Farnesyl phosphoramidothioic acid 6	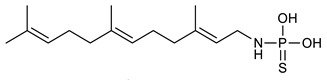	*S. aureus*	Growth inhibition		[127]
9	Arnicolide D	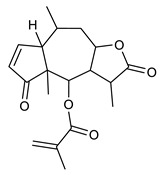	MRSA MSSA	Growth inhibition	Species: *Centipeda minima*	[103]
10	Arnicolide C	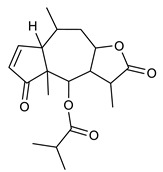	MRSA MSSA	Growth inhibition	Species: *Centipeda minima*	[103]
11	Guaianolide 5	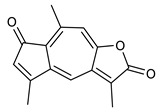	MRSA	Growth inhibition	Species: *Artemisia gilvescens*	[104]
12	Dehydroleucodine	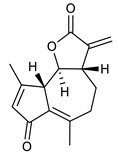	MRSA	Growth inhibition	Species: *Gynoxys verrucosa*	[105]
13	Xanthorrhizol	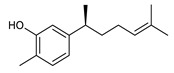	MRSA *E. coli*	Growth inhibition	Species: *Cinnamomum iners*	[106]
**Diterpenes**						
14	Vitexolide A	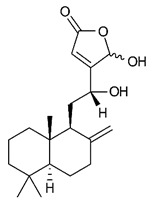	*S. aureus*	Growth inhibition	Species: *Vitex vestita*	[107]
15	Acuminolide	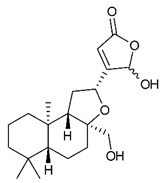	*S. aureus*	Growth inhibition	Species: *Vitex vestita*	[107]
16	12-epivitexolide A	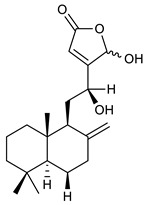	*S. aureus*	Growth inhibition	Species: *Vitex vestita*	[107]
17	8(17),12E,14-labdatrien-6,19-olide	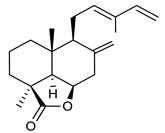	MRSA	Growth inhibition	Species: *Salvia leriifolia*	[108]
18	8(17),11(Z),13(*E*)- trien-15,19-dioic acid	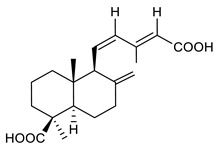	MRSA	Growth inhibition	Species: *Caesalpinia decapetala*	[109]
19	(*E*)-8(17),12-labdadiene-15,16-dial	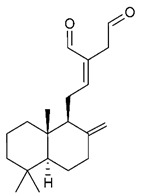	*S. aureus* MRSA	Growth inhibition	Species: *Zingiber montanum*	[110]
20	16α-hydroxycleroda-3, 13 (14)-Z-dien-15, 16-olide (CD)	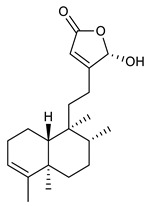	MRSA	Growth inhibition	Species: *Polyathia longifolia*	[128]
21	Coleon U	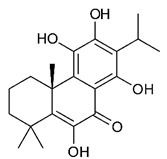	MRSA VRE	Growth inhibition	Species: *Plectranthus grandidentatus Plectranthus hereroensis*	[111]
22	7α-acetoxy-6β-hydroxyroyleanone	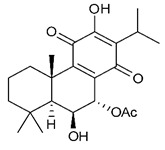	MRSA VRE	Growth inhibition	Species: *Plectranthus grandidentatus Plectranthus hereroensis*	[111]
23	Horminone	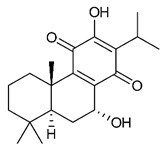	MRSA VRE	Growth inhibition	Species: *Plectranthus grandidentatus Plectranthus hereroensis*	[111]
24	7α,12-dihydroxy-17(15-16)abeo-abieta-8,12,16-triene-11,14-dione	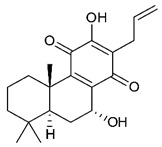	MRSA VRE	Growth inhibition	Species: *Plectranthus grandidentatus Plectranthus hereroensis*	[111]
25	16-Acetoxy-7α,12-dihydroxy-8,12-abietadiene-11,14-dione	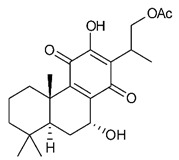	MRSA VRE	Growth inhibition	Species: *Plectranthus grandidentatus Plectranthus hereroensis*	[111]
26	Sincoetsin C	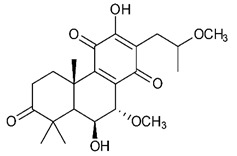	MRSA	Growth inhibition	Species: *Coleus blumei Benth.*	[112]
27	ent-8 (14),15-pimaradien-3β-ol	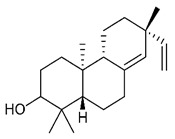	*S. aureus* *S. capitis* *S. haemolyticus* *E. faecalis* *E. epidermidis* *S. pneumoniae*	Cell membrane disruption	Species: *Viguiera arenaria*	[113]
28	rel-15(Ϛ),16-epoxy-7R-hydroxypimar-8,14ene	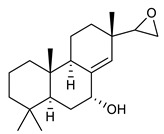	MRSA	Growth inhibition	Species: *Plectranthus ernstii*	[114]
29	rel-15(Ϛ),16-epoxy-7-oxopimar-8,14-ene	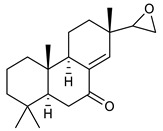	MRSA	Growth inhibition	Species: *Plectranthus ernstii*	[114]
30	1R,11S-dihydroxy-8R,13R-epoxylabd-14-ene	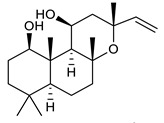	MRSA	Growth inhibition	Species: *Plectranthus ernstii*	[114]
31	Isopimaric acid	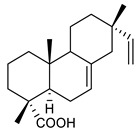	MRSA	Growth inhibition	Species: *Pinus nigra*	[115]
32	ent-kaurenoic acid	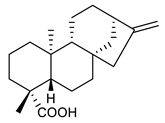	*S. aureus* *S. capitis* *S. epidermidis* *S. haemolyticus* *E. faecalis* MRSA	Cell membrane disruption	Species: *Mikania glomerate* *Citrus reticulate*	[116,117]
33	ent-pimaradienoic acid	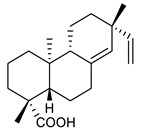	*S. aureus* *S. capitis* *S. epidermidis* *S. haemolyticus* *E. faecalis*	Cell membrane disruption	Species: *Viguiera arenaria*	[116]
34	semisynthetic derivative of 33	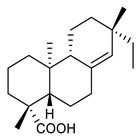	*S. aureus * *S. capitis* *S. epidermidis*	Cell membrane disruption		[116]
35	ent-beyer-15-en-18-O-oxalate (BBN149)	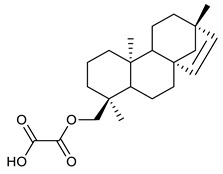	*P. aeruginosa* *K. pneumoniae*	Growth inhibition	Species: *Fabiana densa* var *ramulosa*	[118]
**Triterpenes**						
36	α- Amyrin	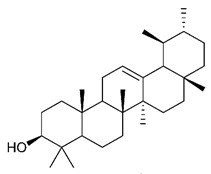	MSSA MRSA	Growth inhibition		[119]
37	Betulinic acid	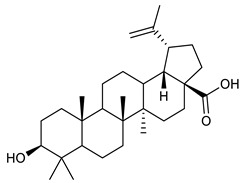	MSSA MRSA	Growth inhibition		[119]
38	Betulinaldehyde	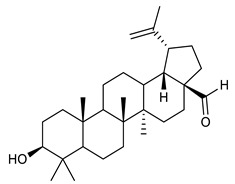	MSSA MRSA	Growth inhibition		[119]
39	Oleanolic acid	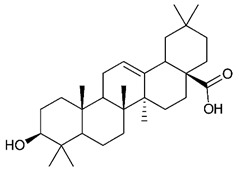	MRSA VRE	Growth inhibition	Species: *Salvia officinalis* (Sage)	[120]
40	Ursolic acid	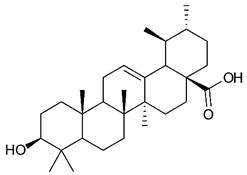	MRSA VRE NASEC NAREC	Growth inhibition	Species: *Salvia officinalis* (Sage) *Eucalyptus tereticornis* *Alstonia scholaris*	[120,121,122]
41	Butyl ester of ursolic acid	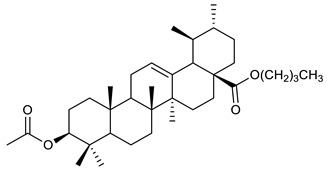	NASEC NAREC	Growth inhibition		[121]
42	Isopropyl ester of ursolic acid	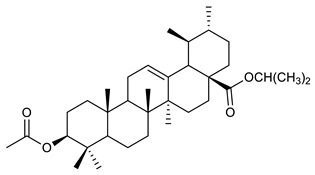	NASEC NAREC	Growth inhibition		[121]
43	18β-glycyrrhetinic acid (18β-GA)	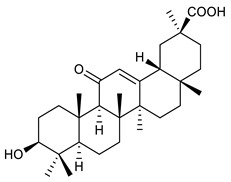	MRSA	Growth inhibition	Species: *Glycyrrhiza glabra*	[131]
44	3β,6β,16β-trihydroxylup-20(29)-ene (CLF1)	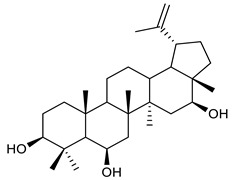	*S. aureus* *E. coli*	Growth inhibition	Species: *Combretum leprosum*	[123]
45	16R-hydroxymollic	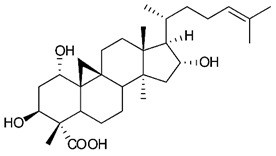	VRE MRSA	Growth inhibition	Species: *Acalypha communis*	[124]
46	15R-hydroxymollic	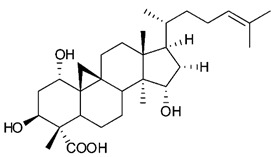	VRE	Growth inhibition	Species: *Acalypha communis*	[124]
47	7β,16β-dihydroxy-1,23-dideoxyjessic acids	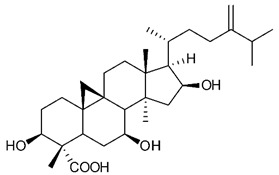	VRE	Growth inhibition	Species: *Acalypha communis*	[124]
48	Aphagrandinoid D	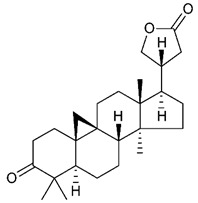	MRSA	Growth inhibition	Species: *Acacia grandifolia*	[125]
49	3β-hydroxylanosta-9,24-dien-21-oic acid	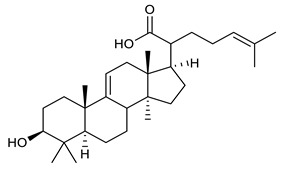	*S. aureus* *P. mirabilis* *Salmonella*	Growth inhibition	Species: *Protorhus longifolia*	[126]
50	methyl-3β-hydroxylanosta-9,24-dien-21-oate	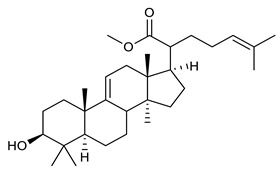	*S. aureus* *P. mirabilis* *Salmonella*	Growth inhibition	Species: *Protorhus longifolia*	[126]



MRSA, methicillin-resistant *S. aureus*; MSSA, methicillin sensitive *S. aureus*; VRE, vancomycin-resistant *Enterococcus faecalis*; NASEC, nalidixic acid-sensitive *E. coli*; NAREC, nalidixic acid-resistant *E. coli*.
